# Using surface-enhanced Raman spectroscopy to probe artificial dye degradation on hair buried in multiple soils for up to eight weeks

**DOI:** 10.1038/s41598-024-57147-2

**Published:** 2024-03-18

**Authors:** Aidan P. Holman, Mackenzi Peterson, Emily Linhart, Dmitry Kurouski

**Affiliations:** 1 Department of Entomology, Texas A&M University, College Station, TX 77843 USA; 2 Department of Biochemistry and Biophysics, Texas A&M University, College Station, TX 77843 USA; 3 Department of Biomedical Engineering, Texas A&M University, College Station, TX 77843 USA; 4 Institute for Advancing Health Through Agriculture, College Station, TX 77843 USA

**Keywords:** Raman spectroscopy, SERS, Hair, Hair dyes, Soils, Optics and photonics, Analytical biochemistry

## Abstract

The discovery of clandestine burials poses unique challenges for forensic specialists, requiring diverse expertise to analyze remains in various states. Bones, teeth, and hair often endure the test of time, with hair particularly exposed to the external environment. While existing studies focus on the degradation of virgin hair influenced by soil pH and decomposition fluids, the interaction between artificial dyes on hair and soil remains underexplored. This paper introduces a novel approach to forensic hair analysis that is based on high-throughput, nondestructive, and non-invasive surface-enhanced Raman spectroscopy (SERS) and machine learning. Using this approach, we investigated the reliability of the detection and identification of artificial dyes on hair buried in three distinct soil types for up to eight weeks. Our results demonstrated that SERS enabled the correct prediction of 97.9% of spectra for five out of the eight dyes used within the 8 weeks of exposure. We also investigated the extent to which SERS and machine learning can be used to predict the number of weeks since burial, as this information may provide valuable insights into post-mortem intervals. We found that SERS enabled highly accurate exposure intervals to soils for specific dyes. The study underscores the high achievability of SERS in extrapolating colorant information from dyed hairs buried in diverse soils, with the suggestion that further model refinement could enhance its reliability in forensic applications.

## Introduction

Seldom does one think of being murdered and buried secretly, only to be found within days, months, or many years, as is encountered in clandestine burials. But for those that are, each scene requires a variety of different forensic specialists depending on the state of the remains. What is typically found, no matter the elapsed time from burial to discovery, are bones, teeth, and hair^1^. While bones and teeth can be concealed by flesh, hair, if not covered by clothing, is usually exposed to the external environment long after death. Studies that work towards forensic hair analysis in these contexts usually measure the degradative effects of soil pH and decomposition fluids on virgin hair^2–4^, but with an increasing trend in dying hair, one may ask how the soil reacts with artificial dyes on hair.

Current forensic hair analysis is primarily based on light microscopy. This technique requires the examination to be carried out by well-trained forensic experts who perform a pattern recognition of between unknown and a library of hair samples^5^. As a result, forensic microscopy of hair is heavily criticized as being too subjective to be used in a court of law, and multiple investigations have been made to re-open former cases that used this type of evidence to support verdicts^6^. In contrast, spectroscopic analyses are very much present and heavily relied upon in forensics for toxicology, geology, and increasingly in pathology^7–9^. One method that’s starting to catch the eyes of forensic professionals is the high-throughput, nondestructive, and non-invasive surface-enhanced Raman spectroscopy (SERS). Surface-enhanced Raman spectroscopy is a technique that amplifies the Raman signal (inelastic scattering of light) by utilizing nanostructured surfaces, providing highly sensitive molecular fingerprinting for substances. In the context of hair, Kurouski and Van Duyne demonstrated how using gold nanoparticles could lead to the strong resonance of colorants found in dyes on dyed hair^10^. Since then, SERS has been used to detect and differentiate over 30 different dyes on hair, as well as probe the degradative effects and still detect the colorant on hair subjected to high heat and weeks of sunlight and lake water exposure^11–14^.

In this study, we employ SERS coupled with partial least squares discriminant analysis (PLS-DA) to assess the efficacy of detecting artificial dyes on hair buried in three distinct soil types for up to eight weeks. Our evaluation also extends to predicting the correct number of weeks since burial based on SERS spectra. This valuable information not only solidifies the role of SERS in forensic hair analysis but may also hold the potential to provide insights into post-mortem intervals.

## Materials and methods

### Hair preparation

The hair used in this research remained unchanged, consisting of natural, untreated hair from a voluntarily donating 21-year-old Caucasian female. She was thoroughly briefed on the intended use of her hair. The hair was dyed using Ion brand hair dye either of Ion Jet Black (permanent black—“PBA”), Ion Sapphire (permanent blue—“PBU”), Ion Radiant Orchid (permanent purple—“PPU”), Ion Garnet (permanent red—“PRD), Ion Blackest Black (semi-permanent black—“SBA”), Ion Sapphire (semi-permanent blue—“SBU”), Ion Radiant Orchid (semi-permanent purple—“SPU”), or Ion Garnet (semi-permanent red—“SRD”). The information for each hair dye and colorants can be found in the SI, Tables S1 and S2. A clean beaker was used to mix permanent hair dye and activator and a clean graduated cylinder was used to pour equal portions of each hair colorant onto each batch of hair. Permanent dyes were mixed with an Ion Sensitive Scalp Creme Developer in volume ratios consistent with the manufacturer’s instruction label. The colorant was then gently rubbed in until all hair strands in each batch were completely coated. After the elapsed time indicated by the instruction label on the box passed, the hair was rinsed off under low pressure deionized water within a small stainless-steel strainer until the water running off was clear, after which the hair was left to air dry. All experiments were performed in accordance with NIH guidelines and regulations.

Previously reported study by our group showed that SERS could be used to distinguish hair from different races/ethnicities, age groups, and sexes^15^. Therefore, the reported results should be valid only for young female individuals of Caucasian race. In following studies, we expect to expand the reported results to other races/ethnicities, age groups, and genders. However, the results of these studies are the subjects for separate studies.

### Experimental treatment

Cactus, Palm & Citrate Potting Mix (The Scotts Miracle-Gro Company, Oregon, US) (henceforth, Soil Type A or “STA”), All Purpose Garden Soil (The Scotts Miracle-Gro Company, Oregon, US) (henceforth, Soil Type B or “STB”), and clay soil (collected from Bryan, Texas) (henceforth, Soil Type C or “STC”) were all used as soil types for this experiment. The pH of each soil type was measured using a benchtop pH meter to be 6.38, 6.81, and 9.96, for soil types A, B, and C, respectively. In our previous study, we showed that substances with acidic pH, such as white wine and orange juice, drastically lower the accuracy of SERS-based identification of colorants on hair^16^. We hypothesized that the acidic pH facilitates the desorption of artificial colorants from the hair surface. Therefore, we reported pHs of the soils used in our study. Based on the reported pHs, we can conclude that none of the analyzed soils were powerfully acidic. As a result, no acid-driven degradation of colorants should be expected.

Soil was placed outside within ten-gallon pots, filled to an inch below the top, to endure realistic weather conditions within the College Station-Bryan area. The dyed hair was placed completely under the soil (but no more than 5 cm) so that none of the dyed hair was exposed directly to sunlight or other weather conditions.

### Sample collection

Samples were buried in respective soils for a full week before each collection. During collection, hair groups were removed all-at-once from their soil to snip an inch from a few hair strands that were then sealed in a plastic bag and stored in a dark environment to be later used for scanning/analysis. Removed hair groups were then re-buried in the same position they were removed. The collection of samples stopped after eight weeks of exposure to the respective soils.

### Raman spectroscopy

The gold nanoparticle (AuNP) solution, excitation wavelength of laser light, equipment, and power were chosen based on published methods from Esparza and coworkers^17^. The AuNPs were characterized using JEOL JSM-7500F Scanning Electron Microscopy (SEM) and SERS, Figs. S1, S2. SERS spectra were collected using a TE-2000U Nikon inverted confocal microscope, equipped with a 20 × objective. A solid-state laser generated 785 nm light, while power through each sample was kept at 1.8 mW. Scattered light was collected using the same magnification and directed using a 50/50 beam splitter into an IsoPlane-320 spectrometer (Princeton Instruments) equipped with a 600 groove/mm grating. Prior to entering the spectrometer, elastically scattered photons were blocked by a long-pass filter (Semrock, LP03-785RS-25). Inelastically scattered photons were collected using PIX-400BR CCD (Princeton Instruments).

Fifty spectra from each sample, comprising 15–20 spectra from each of three regions on one hair strand per sample group, were collected by placing each hair on a glass cover slide and applying ~ 5 µL of the AuNP solution described above. Hairs within groups that consistently returned spectra with full AuNP signature contribution were only scanned five times for the relevant sampling period. [Note: The absence of colorant molecules for proper spectral acquisition of these colorants can be attributed to a possible larger predisposition to degradation within certain soils This susceptibility is likely influenced by the exclusive reliance on a single primary colorant compound, 1-Hydroxyethyl-4,5-Diamino Pyrazole, in both pertinent dyes (PPU and PRD). Additionally, while PBA shares this compound, it incorporates another colorant, Toluene-2,5-Diamine.] The strand of hair was coated by the 5 µL drop AuNP solution by moving the hair around the slide until the nanorod solution outlined ~ 10 mm in length (incidental of whether the strand was longer or shorter than 10 mm) of the strand of hair. The laser light was positioned on the hair medial-proximally, as had the most consistently intense peaks for bands of interest. Overall acquisition times ranged from 18 to 30 s.

### Reference dye preparation

In order to extrapolate the (expected) source of the primary molecules responsible for resonances seen within spectra, the respective hair dyes for each group were analyzed without reaction to hair. To do so, 50 mg of each semi-permanent dye was placed on a glass coverslip and mixed with 5 uL of AuNPs. For permanent hair dyes, 50 mg of each dye was mixed 500 uL of developer, mixed by hand until homogenous and left to oxidize overnight. Once oxidized (i.e., the solution appeared to be its expected color), 50 uL of each activated dye was placed on a glass coverslip and 5 uL of AuNPs were added. All mixtures were analyzed with the same instrument above at 12–20 s acquisition times using 8 mW (785 nm) laser power.

### Data analysis

All spectra were trimmed from 308 cm^−1^ to 1708 cm^−1^ (for noise reduction in analyses), smoothed (2nd order) using a Savitzky–Golay filter, baseline-corrected (2nd order) using automatic weighted least squares, and area-normalized (all data points of all spectra were normalized to their respective bands) before analysis using MATLAB (as displayed). Chemometric analysis of acquired spectra was done in MATLAB equipped with PLS_Toolbox 9.0 (Eigenvector Research, Inc., Manson, WA). For PLS-DA, cross-validations from full calibration models were employed unless stated otherwise. Pre-processing of each ANOVA graph and PLS-DA model was done using mean centering and 1st-derivative smoothing (n = 2, fl = 15 pt.). The number of latent variables (LVs) (loadings plots can be found in Figs. S3–S6) per model were selected based on the most appropriate root mean square error (in cross-validation) value for each model.

## Results and discussion

**Figure 1:**
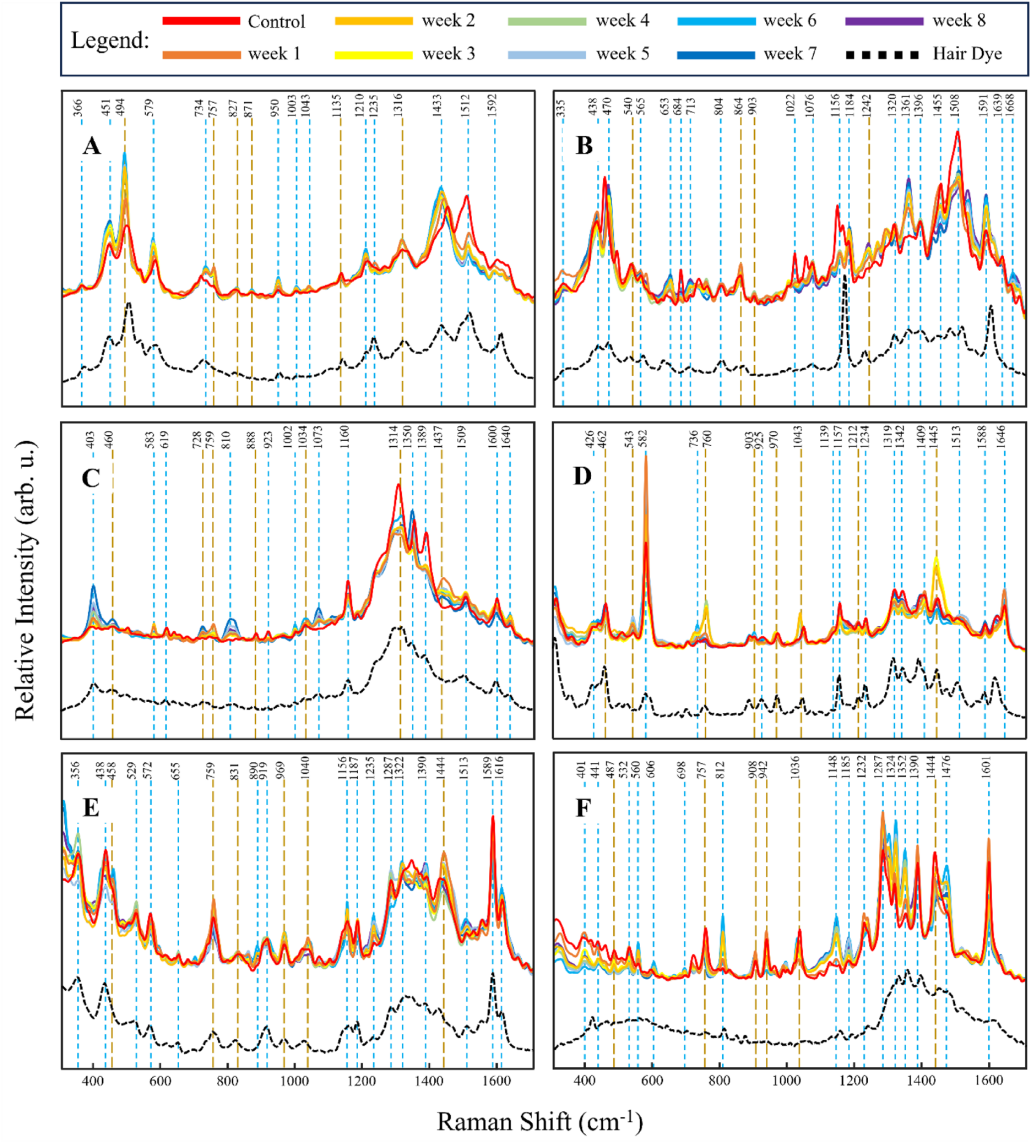
Averaged SERS spectra from hair colored with (**A**) PBA, (**B**) PBU, (**C**) SBA, (**D**) SBU, (**E**) SPU, and (**F**) SRD, buried in combined soils (Soil types **A**–**C**) with corresponding dye signatures. Gold dashed lines represent bands likely promoted by AuNPs and blue dashed lines represent all other bands from the colorant.

**Figure 2:**
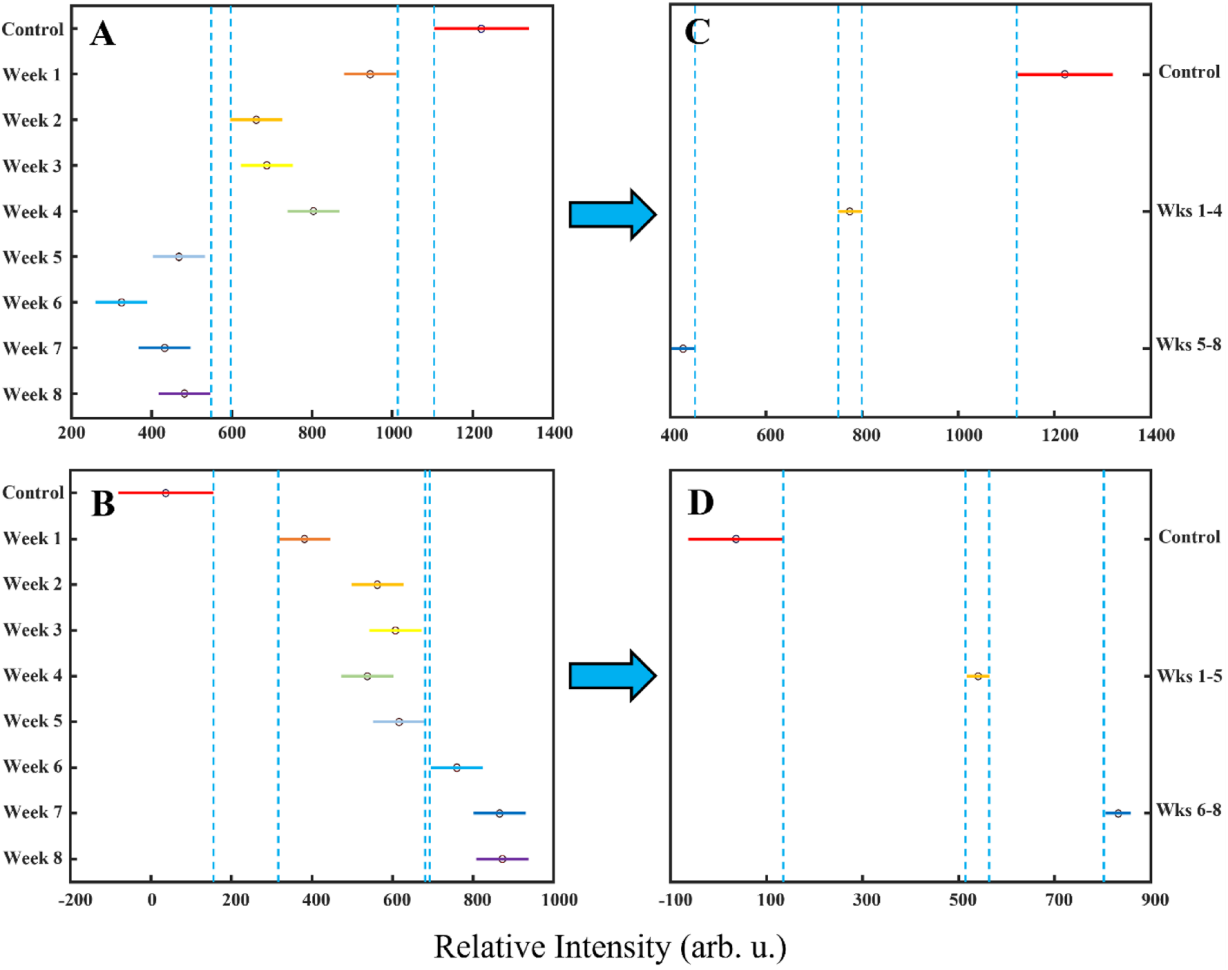
Kruskal–Wallis ANOVA multiple comparison graphs of relative intensities in spectra acquired from hair dyed with (**A**) PBA at 1512 cm^−1^ and (**B**) PBU at 1361 cm^−1^, including after combining class groups for (**C**) PBA and (**D**) PBU, respectively. The solid-colored bars represent 95% confidence intervals for each class (control and weeks), the black circles represent mean values, and the blue dashed lines represent classification thresholds, determined by relevant confidence intervals.

In the SERS spectra acquired from PBA-dyed hair before placement in soils (control), we detected vibrational bands centered at 366, 451, 494, 579, 734, 757, 827, 871, 950, 1003, 1043, 1135, 1210, 1235, 1316, 1433, 1512, and 1592 cm^–1^, Fig. 1 and Table S3. As time progressed, we observed a drastic change between the relative intensities of 1512 and 1433 cm^−1^ band peaks, corresponding to C–C stretching likely from aromatic rings of the colorants, attributing to the degradative effects of soils on PBA over time. Because the most notable difference is in the relative intensities of peaks centered at 1512 cm^−1^, we performed ANOVA of PBA spectra at this peak, Fig. 2. While the initial pattern of degradation is not apparently linear and well-distinguished, when time exposed groups were combined based on relative intensity range similarities (within blue dashed lines, Fig. 2), a more unique and linear model is given.

In the SERS spectra acquired from PBU-dyed hair before placement in soils, we detected vibrational bands centered at 335, 438, 470, 540, 565, 653, 684, 713, 804, 864, 903, 1022, 1076, 1156, 1184, 1242, 1320, 1361, 1396, 1455, 1508, 1591, 1639, and 1668 cm^–1^, Fig. 1 and Table S3. As time progressed, we see a strong change between the relative intensities of peaks at 1361 cm^−1^, for example, which also corresponds to C–C stretching from the aromatic rings of the colorants. Because of this, we performed ANOVA of PBU spectra at this band and found an overall strong positive correlation between peak height and exposure time. We further sought to increase linearity and uniqueness of groups by grouping weeks with similar relative intensity ranges together.

In the SERS spectra acquired from PPU-dyed hair before placement in soils, we detected vibrational bands centered at 382, 456, 508, 567, 642, 663, 726, 761, 851, 903, 942, 965, 1003, 1040, 1100, 1131, 1190, 1236, 1270, 1348, 1442, 1484, and 1589 cm^−1^, Fig. S7 and Table S3. It should be noted that beyond control, the spectra became almost entirely reflective of spectra from AuNPs. The same can be said for PRD, for which the control spectra had peaks at vibrational bands centered at 419, 456, 545, 581, 726, 761, 943, 965, 1003, 1040, 1100, 1131, 1190, 1235, 1299, 1320, 1348, and 1442 cm^−1^, Fig. S7 and Table 2. ANOVA was carried out for both colorants using peaks from scattering from AuNPs and, unsurprisingly, no clear linearity could be derived, Fig. S8.

**Figure 3:**
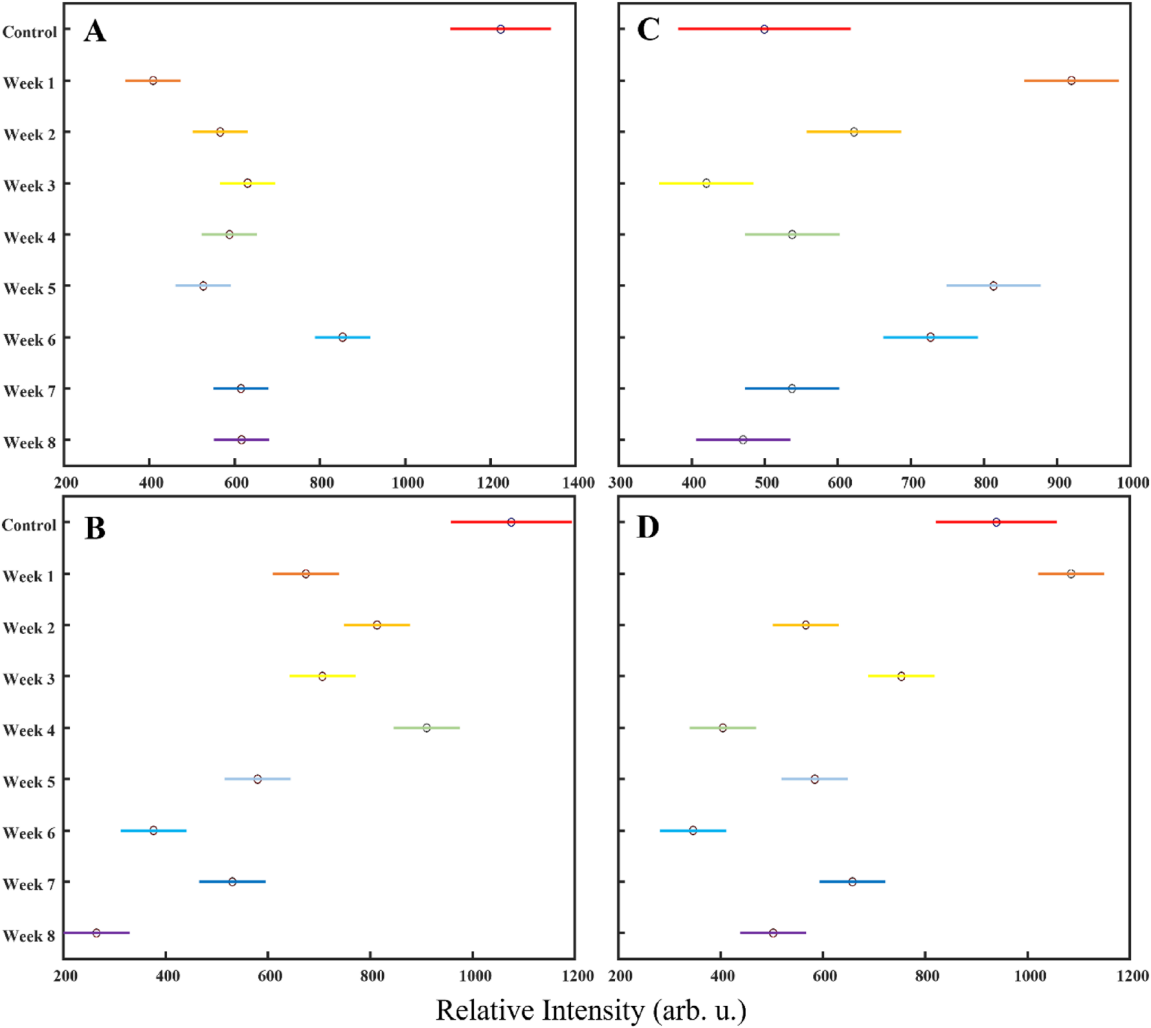
Kruskal–Wallis ANOVA multiple comparison graphs of relative intensities in spectra acquired from hair dyed with (**A**) SBA at 1314 cm^−1^, (**B**) SBU at 582 cm^−1^, (**C**) SPU at 1588 cm^−1^, and (**D**) SRD at 1601 cm^−1^. The solid-colored bars represent 95% confidence intervals for each class and the black circles represent mean values.

In the SERS spectra acquired from SBA-dyed hair before placement in soils, we detected vibrational bands centered at 403, 460, 583, 619, 728, 759, 810, 888, 923, 1002, 1034, 1073, 1160, 1314, 1350, 1389, 1437, 1509, 1600, and 1640 cm^−1^, Fig. 1 and Table S3. From SBU-dyed hair, we detected vibrational bands at 426, 462, 543, 582, 736, 760, 903, 925, 970, 1043, 1139, 1157, 1212, 1234, 1319, 1342, 1409, 1445, 1513, 1588, and 1646 cm^−1^, Fig. 1 and Table S3. From SPU-dyed hair, we detected vibrational bands at 356, 438, 458, 529, 572, 655, 759, 831, 890, 919, 969, 1040, 1156, 1187, 1235, 1287, 1322, 1390, 1444, 1513, 1589, and 1616 cm^−1^, Fig. 1 and Table S3. Finally, from SRD-dyed hair, we detected vibrational bands at 401, 441, 487, 532, 560, 606, 698, 757, 812, 908, 942, 1036, 1148, 1185, 1232, 1287, 1324, 1352, 1390, 1444, 1476, and 1601 cm^−1^, Fig. 1 and Table S3. ANOVA was also performed for these groups’ spectra and no distinguishable intensity-related groupings were made, Fig. 3. In light of the absence of apparent linearity and well-distinguished groupings, we cannot say that one peak can be used to assess degradation for these colorants, but potentially multiple peaks used in unison are more appropriate for the identification of exposure time.

Raman bands peaking either higher or lower than the control spectra, and exhibiting slight fluctuations over time, may indicate colorants with higher susceptibility to degradation. The analysis of such bands contributes to enhanced fluorescence, revealing the remaining colorant during examination (Table 1).

**Table 1:** Assignments for Raman bands found in cyclic compounds: ν, stretching; δ, in-plane bending; π, out-of-plane bending.

Raman Band (s) (± 5 cm^−1^)	Assignment
380–450^18, 19^	π (N=N)/ π (C–N)
420–480^20^	π (C–C)
540^20^	δ (C–C)
700–790^19^	π (N–H)
810–840^19^	ν (C–C)
915–950^19^	ν (N–C)
1010–1050^19^	ν (N=N)
1100–1260^20^	v (O–H)
1100–1450^20^	v (C–O)
1250–1310^18^	ν (N–C)
1400–1650^20^	v (C=C–C) (aryl/phenyl ring)
1580–1600^18^	δ (N–H)

Without an obvious extrapolation of a post-burial interval from the ANOVA results, we considered the use of machine learning, which instead of analyzing the variance at one peak, a model can be built using all qualities of multiple peaks by week per colorant. Therefore, we utilized PLS-DA to determine the accuracy of SERS-based identification of exposure time for hair buried in varying soils for up to 8 weeks, Table 2. We found that control and week one samples (from all soils) allowed for the best differentiation by PLS-DA, with overall accuracies of 99.25 and 85.42%, respectively. PPU and PRD colorants contributed the overall greatest differentiations between weeks with 78.52 and 88.89%, respectively. However, the reasoning for this is likely due to the full resonance contribution of the AuNPs used as discussed earlier as well as the relatively low number of spectra acquired compared to other dye groups.

**Table 2:** Full calibration PLS-DA confusion matrices results of each dye for each week, featuring the true positive rate (TPR) indicating the accuracy of the program to predict that length of exposure time among the rest and the overall success rate (OSR) of the program’s ability to predict all weeks per colorant.

Dye (LV#)	OSR (%)	Control (TPR%) (n = 50)	Predicted Exposure Time True Positive Rates (%)							
			1 Week (n = 150)	2 Weeks (n = 150)	3 Weeks (n = 150)	4 Weeks (n = 150)	5 Weeks (n = 150)	6 Weeks (n = 150)	7 Weeks (n = 150)	8 Weeks (n = 150)
PBA (7)	59.04	100	98	58.67	59.33	56	18.67	61.33	2.67	76.67
PBU (6)	54.82	100	89.33	16.67	44.67	80	18	78.67	42.67	23.33
PPU (6)	78.52	100	100	66.67	66.67	60	26.67	93.33	93.33	100
PRD (6)	88.89	100	100	93.33	86.67	93.33	93.33	73.33	80	80
SBA (6)	53.7	100	66	56.67	47.33	0	49.33	48.67	46	69.33
SBU (7)	68.15	100	86	46	12.67	58.67	69.33	86	66.67	88
SPU (7)	57.7	100	56	38.67	13.33	50	40.67	46	83.33	91.33
SRD (7)	62.15	94	88	24.67	44	9.33	72.67	88	48.67	90
OSR (%)	65.37	99.25	85.42	50.17	46.83	50.92	48.58	71.92	57.92	77.33

OSR = Mean TPR%.

From the modified groups in the ANOVA analysis of PBA and PBU shown in Fig. 2, we wanted to explore if PLS-DA improved at identifying the time exposed, Table 3. We found that PLS-DA allowed for the elucidation of exposed time of PBA-dyed hair for control with 100% accuracy, combined one-to-four weeks buried with 92.33% accuracy, and combined five-to-eight weeks buried with 91.5% accuracy. What’s more is PLS-DA allowed for the elucidation of exposed time of PBU-dyed hair for control with 100% accuracy, combined one-to-five weeks buried with 95.47% accuracy, and combined six-to-eight weeks buried with 95.11% accuracy. This indicates that while most exposure times could not be well extrapolated from most dyes for combined soils, specific dyes may allow for an accurate range of weeks determination.

**Table 3:** Full calibration PLS-DA confusion matrices for combined time exposed groups from Fig. 2.

Dye	TPR (%)	Predicted class	Actual class		
			Control (n = 50)	1–4 Weeks (n = 600)	5–8 Weeks (n = 600)
PBA	100	Control	50	15	0
	92.33	1–4 Weeks	0	554	51
	91.5	5–8 Weeks	0	31	549

Dye	TPR (%)	Predicted class	Control (n = 50)	1–5 Weeks (n = 750)	6–8 Weeks (n = 450)
PBU	100	Control	50	0	0
	95.47	1–5 Weeks	0	716	22
	95.11	6–8 Weeks	0	34	428

One may ask how drastic the individual effects are from each soil on detecting each colorant. For this we calibrated (trained) a PLS-DA model with the control colorants spectra and validated (tested) the model using spectra separated by soil type, Table 4. We found that STA had the least drastic effects on PBA, PBU, SBU, SPU, and SRD with TPRs of 100, 100, 99.75, 92, and 100%, respectively. STA’s ability to extrapolate hair dyed with PPU, PRD, and SBA, was poor with TPRs of 20, 37.5, and 6.5%, respectively. Additionally, we found that STB had small effects on PBA, PBU, SBU, SPU, and SRD with TPRs of 99.25, 100, 100, 98, and 99.75%, respectively. The effects of STB on PPU, PRD, and SBA dyed hair were shown to be significant with TPRs of 7.5, 47.5, and 6%, respectively. Finally, dyed hair buried in STC still allowed for highly accurate identification of PBA, PBU, SBU, SPU, and SRD groups, with TPRs of 100, 100, 99.75, 92, and 100%, respectively. Just as with STA and STB, STC had apparently large effects on hair dyed with PPU, PRD, and SBA, given TPRs of 20, 37.5, and 6.5%, respectively.

**Table 4:** Control-calibrated PLS-DA confusion matrices results for prediction models tested using spectra grouped by soil type.

	OSR (%)	Predicted dye true positive rates (%)							
		PBA	PBU	PPU	PRD	SBA	SBU	SPU	SRD
Calibrated soil (LV#)									
All soils (6)	99.75	100	100	98	100	100	100	100	100
Validated soil (LV#)									
STA (6)	69.47	100	100	20	37.5	6.5	99.75	92	100
STB (6)	69.75	99.25	100	7.5	47.5	6	100	98	99.75
STC (6)	68.67	87.5	99	32.5	37.5	0	93	99.75	100
OSR (%)	69.3	95.58	99.67	20	40.83	4.17	97.58	96.58	99.92

While the TPR results of PPU and PRD should be of no surprise since the spectra beyond control are quite different than the control, PLS-DA of SBA-dyed hair buried in each soil type consistently generated poor results. Upon further analysis into the reason for which this was occurring, it was noted that most spectra from SBA-dyed hair past control were predicted as belonging to PBA-dyed hair spectra. This indicates that the colorants between PBA and SBA are very similar, which is known^10^, and in order to build a more reliable PLS-DA model, more spectra from SBA, after different environmental effects such as in this experiment are applied, should be uploaded for model calibration to cover more grounds, which is demonstrated in the full calibration model from all soils for all colorants, Table 5. Using spectra from all degradative time points, PLS-DA was still able to differentiate all models (save PPU and PRD) with over 90% accuracy, including SBA which jumped from an overall 4.17% prediction accuracy to a 98.24% prediction accuracy in this model. These results indicate an overall high achievability of SERS to extrapolate colorant information of dyed hairs buried in varying soils.

**Table 5:** Full calibration PLS-DA confusion matrix for all colorants from all soils and controls.

Predicted dye	TPR (%)	Actual dye							
		PBA (n = 1250)	PBU (n = 1250)	PPU (n = 170)	PRD (n = 170)	SBA (n = 1250)	SBU (n = 1250)	SPU (n = 1250)	SRD (n = 1250)
PBA	97.36	1217	0	13	0	0	0	0	0
PBU	98.96	0	1237	0	0	0	0	0	0
PPU	1.76	0	0	3	71	20	0	0	3
PRD	0.59	3	1	89	1	2	17	4	11
SBA	98.24	30	12	65	98	1228	0	0	9
SBU	92.88	0	0	0	0	0	1161	0	0
SPU	98.08	0	0	0	0	0	72	1226	0
SRD	98.16	0	0	0	0	0	0	20	1227

## Conclusions

In conclusion, the SERS spectra analysis of dyed hair exposed to different soils reveal the progressive degradation of permanent and semi-permanent colorants over time. The significant changes observed in the relative intensities of specific vibrational bands, particularly at 1512 cm^−1^ for PBA and 1361 cm^−1^ for PBU, suggest the impact of soil exposure on dye degradation. Despite the absence of clear linearity and well-distinguished groupings in the ANOVA results for some colorants, the application of PLS-DA proved to be a promising approach for accurate identification of exposure time. Notably, the control and one-week buried samples exhibited the best differentiation from all spectra and dyes, emphasizing the potential of SERS in determining post-burial intervals. Additionally, the individual effects of different soils on each colorant were assessed, revealing varying levels of sensitivity. While challenges were identified, such as the similarity between PBA and SBA spectra, the overall high achievability of SERS in extrapolating colorant information from dyed hairs buried in diverse soils is evident. Further refinement of models, particularly with more spectra from SBA under environmental effects, could enhance the reliability of SERS-based assessments in forensic applications. Additionally, future studies involving the effects of decomposition fluids in unison with soil would significantly improve the comparability of dyed hair analysis for hair collected from clandestine burials.

### Supplementary Information

Supplementary Information.

## Data Availability

The datasets used and/or analyzed during the current study available from the corresponding author on reasonable request.
