# Supplementary material for: Using surface-enhanced Raman spectroscopy to probe artificial dye degradation on hair buried in multiple soils for up to eight weeks
Source: Sci Rep. 2024 Mar 18;14:6469. doi: 10.1038/s41598-024-57147-2 (PMC10948827; doi:10.1038/s41598-024-57147-2)
Supplement: Supplementary file 1 — Supplementary Information. [file 41598_2024_57147_MOESM1_ESM.docx]

Using Surface-Enhanced Raman Spectroscopy to Probe Artificial Dye Degradation on Hair Buried in Multiple Soils for Up to Eight Weeks

Aidan P. Holman,^1-2^ Mackenzi Peterson,^1-2^ Emily Linhart,^1^ and Dmitry Kurouski^2-4*^

*E-mail: dkurouski@tamu.edu Tel: 979-458-3778.

ORCID

Aidan Holman: 0000-0003-4244-7348

Dmitry Kurouski: 0000-0002-6040-4213

1. Department of Entomology, Texas A&M University, College Station, Texas 77843, United States
2. Department of Biochemistry and Biophysics, Texas A&M University, College Station, Texas 77843, United States
3. Department of Biomedical Engineering, Texas A&M University, College Station, Texas, 77843, United States
4. Institute for Advancing Health through Agriculture, College Station, Texas, 77843, United States

Supporting Information

**Table S1.** Hair Dye Identifiers, respective groups classifications and colorant(s), as well as coupler compounds that bind to the colorant(s) during oxidation, where applicable.

| **Hair Dye Item (SBS-######)** | **Group** | **Colorant(s) (*and Couplers*) in Dyes** |
| --- | --- | --- |
| Ion Jet Black (305430) | PBA | (1) 2,4-Diaminophenoxyethanol (*coupler*), (2) Toluene-2,5-Diamine, and (3) 1-Hydroxyethyl-4,5-Diamino Pyrazole |
| Ion Sapphire (405601) | PBU | (1) 5-Amino-6-Chloror-o-Cresol (*coupler*) and (2) N,N-Bis(2-Hydroxyethyl)-p-phenylenediamine |
| Ion Radiant Orchard (405611) | PPU | (1) 2,6-Diaminopyridine (*coupler*) and (2) 1-Hydroxyethyl-4,5-Diamino Pyrazole |
| Ion Garnet (405602) | PRD | (1) Hydroxyethyl-3,4-Methylenedioxyaniline (*coupler*) and (2) 1-Hydroxyethyl-4,5-Diamino Pyrazole |
| Ion Blackest Black (405079) | SBA | (1) Basic Blue 99, (2) Basic Brown 16, (3) HC Blue No. 2, and (4) HC Yellow No. 4 |
| Ion Sapphire (405068) | SBU | (1) Basic Yellow 87, (2) Basic Blue 124, and (3) HC Blue No. 15 |
| Ion Radiant Orchard (405045) | SPU | (1) Basic Violet 2 and (2) HC Blue No. 15 |
| Ion Garnet (405066) | SRD | (1) Basic Red 51, (2) Basic Yellow 87, and (3) HC Blue No. 15 |

**Table S2.** Colorant and its respective two-dimensional structure for its active (oxidized when relevant) form.

| **Colorant (permanence found in)** | **Active 2-D Structure*** |
| --- | --- |
| Basic Blue 99 (semi-permanent) | 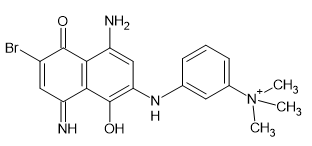 |
| Basic Blue 124 (semi-permanent) | 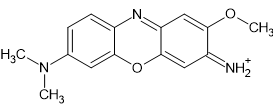 |
| Basic Brown 16 (semi-permanent) | 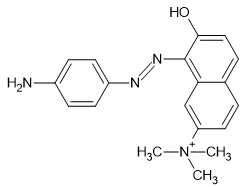 |
| Basic Red 51 (semi-permanent) | 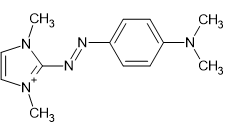 |
| Basic Yellow 87 (semi-permanent) | 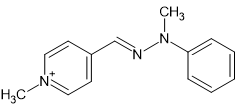 |
| Basic Violet 2 (semi-permanent) | 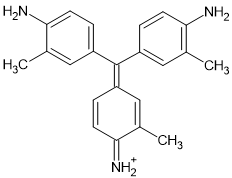 |
| HC Blue No. 2 (semi-permanent) | 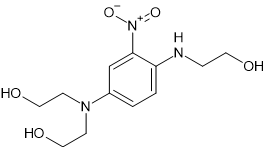 |
| HC Blue No. 15 (semi-permanent) | 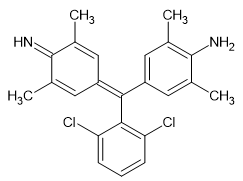 |
| HC Yellow No. 4 (semi-permanent) | 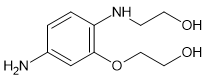 |
| Toluene-2,5-Diamine (permanent) | 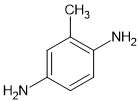 |
| 2,4-Diaminophenoxyethanol (permanent) | 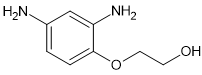 |
| 1-Hydroxyethyl-4,5-Diamino Pyrazole (permanent) | 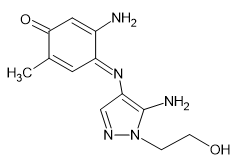 |
| 5-Amino-6-Chloror-o-Cresol (permanent) | 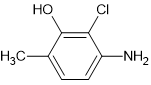 |
| N,N-Bis(2-Hydroxyethyl)-p-phenylenediamine (permanent) | 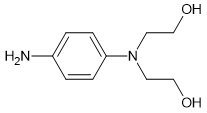 |
| 2,6-Diaminopyridine (permanent) | 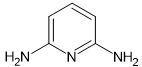 |
| Hydroxyethyl-3,4-Methylenedioxyaniline (permanent) | 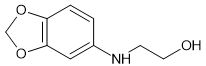 |

**All 2-D structure images were made in ACD/ChemSketch (Freeware) (Advanced Chemistry Development, Inc.).*

**Table S3.** Vibrational bands in the Raman spectra acquired from the control samples and AuNPs.

|  | **Vibrational Bands (cm^-1^)** |
| --- | --- |
| **AuNPs** | 461, 492, 540, 672, 726, 761,829, 869, 903, 942, 965, 1039, 1066, 1096, 1130, 1198, 1216, 1247, 1267, 1311, 1376, 1442 |
| **Dye** | **Vibrational Bands (cm^-1^)** |
| **PBA** | 366, 451, 494, 579, 734, 757, 827, 871, 950, 1003, 1043, 1135, 1210, 1235, 1316, 1433, 1512, 1592 |
| **PBU** | 335, 438, 470, 540, 565, 653, 684, 713, 804, 864, 903, 1022, 1076, 1156, 1184, 1242, 1320, 1361, 1396, 1455, 1508, 1591, 1639, 1668 |
| **PPU** | 382, 456, 508, 567, 642, 663, 726, 761, 851, 903, 942, 965, 1003, 1040, 1100, 1131, 1190, 1236, 1270, 1348, 1442, 1484, 1589 |
| **PRD** | 419, 456, 545, 581, 726, 761, 943, 965, 1003, 1040, 1100, 1131, 1190, 1235, 1299, 1320, 1348, 1442 |
| **SBA** | 403, 460, 583, 619, 728, 759, 810, 888, 923, 1002, 1034, 1073, 1160, 1314, 1350, 1389, 1437, 1509, 1600, 1640 |
| **SBU** | 426, 462, 543, 582, 736, 760, 903, 925, 970, 1043, 1139, 1157, 1212, 1234, 1319, 1342, 1409, 1445, 1513, 1588, 1646 |
| **SPU** | 356, 438, 458, 529, 572, 655, 759, 831, 890, 919, 969, 1040, 1156, 1187, 1235, 1287, 1322, 1390, 1444, 1513, 1589, 1616 |
| **SRD** | 401, 441, 487, 532, 560, 606, 698, 757, 812, 908, 942, 1036, 1148, 1185, 1232, 1287, 1324, 1352, 1390, 1444, 1476, 1601 |





**Figure S1.** SEM image of prepared AuNPs.


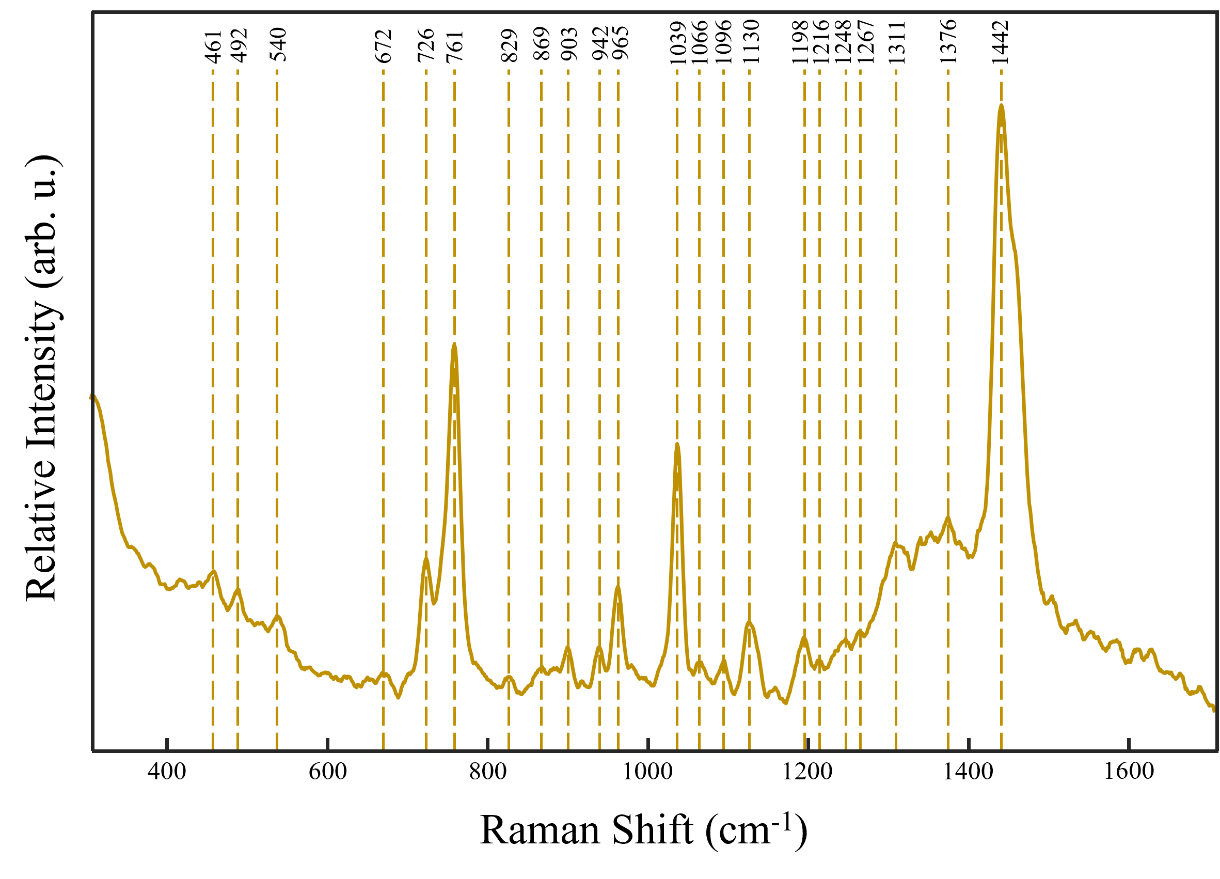


**Figure S2.** SERS spectra of AuNPs (on glass coverslip).


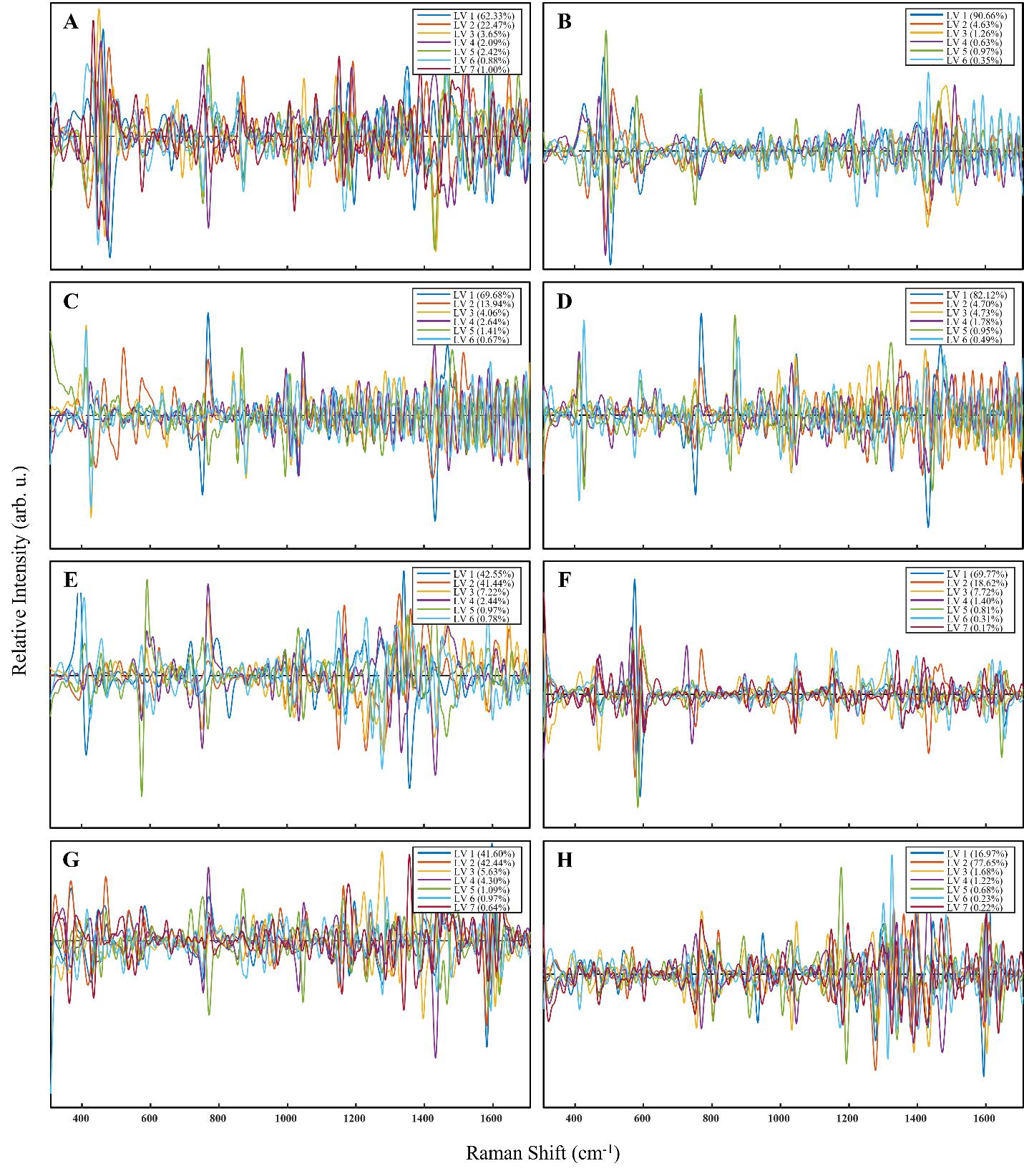


**Figure S3.** PLS-DA loadings plots for latent variables within collective (A) PBA-, (B) PBU-, (C) PPU-, (D) PRD-, (E) SBA-, (F) SBU-, (G) SPU-, and (H) SRD-dyed hair spectra for results used in Table 2.

**
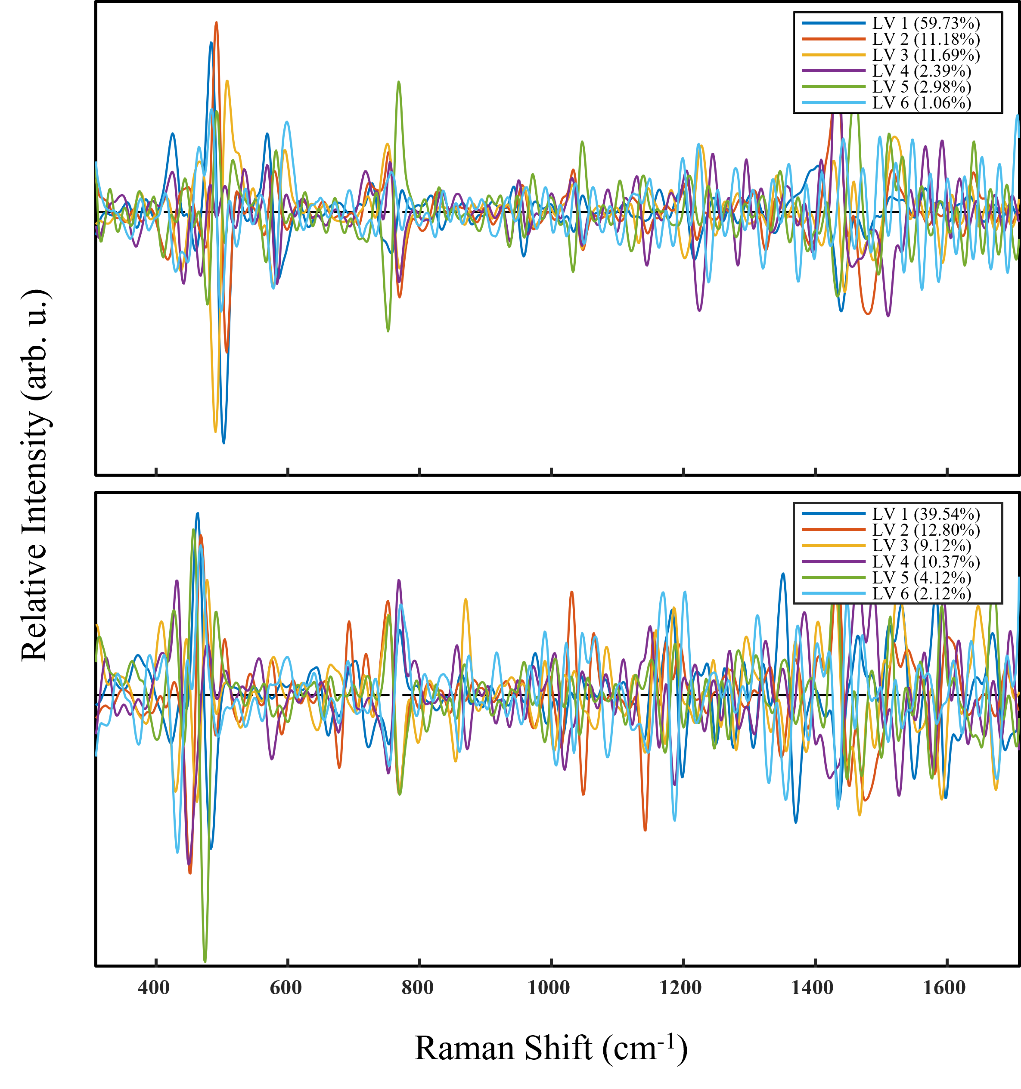
**

**Figure S4.** PLS-DA loadings plots for latent variables within collective PBA-(top) and PBU-dyed hair spectra (bottom) with modified groups of weeks for results used in Table 3.


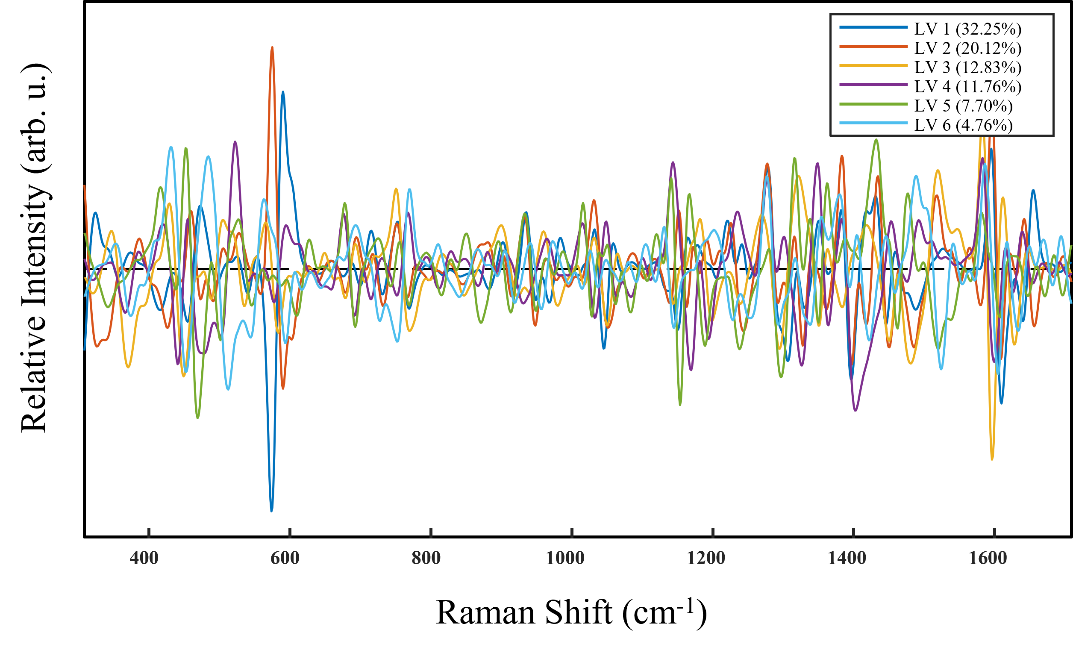


**Figure S5.** PLS-DA loadings plot for latent variables for control for results used in Table 4.


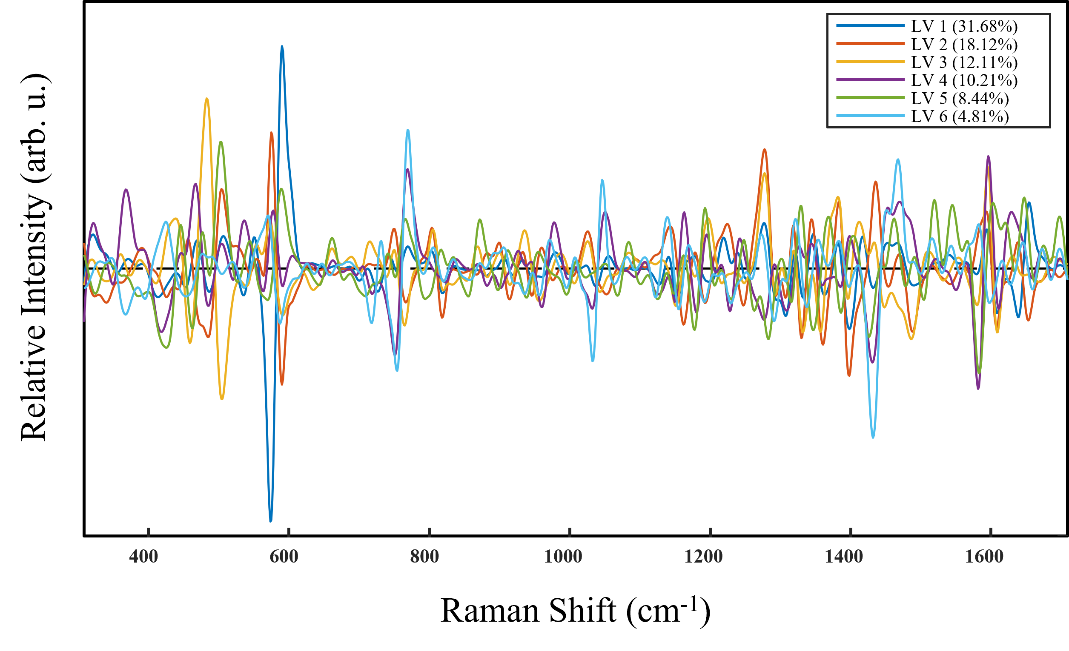


**Figure S6**. PLS-DA loadings plot for latent variables within all dyes spectra for results used in Table 5.


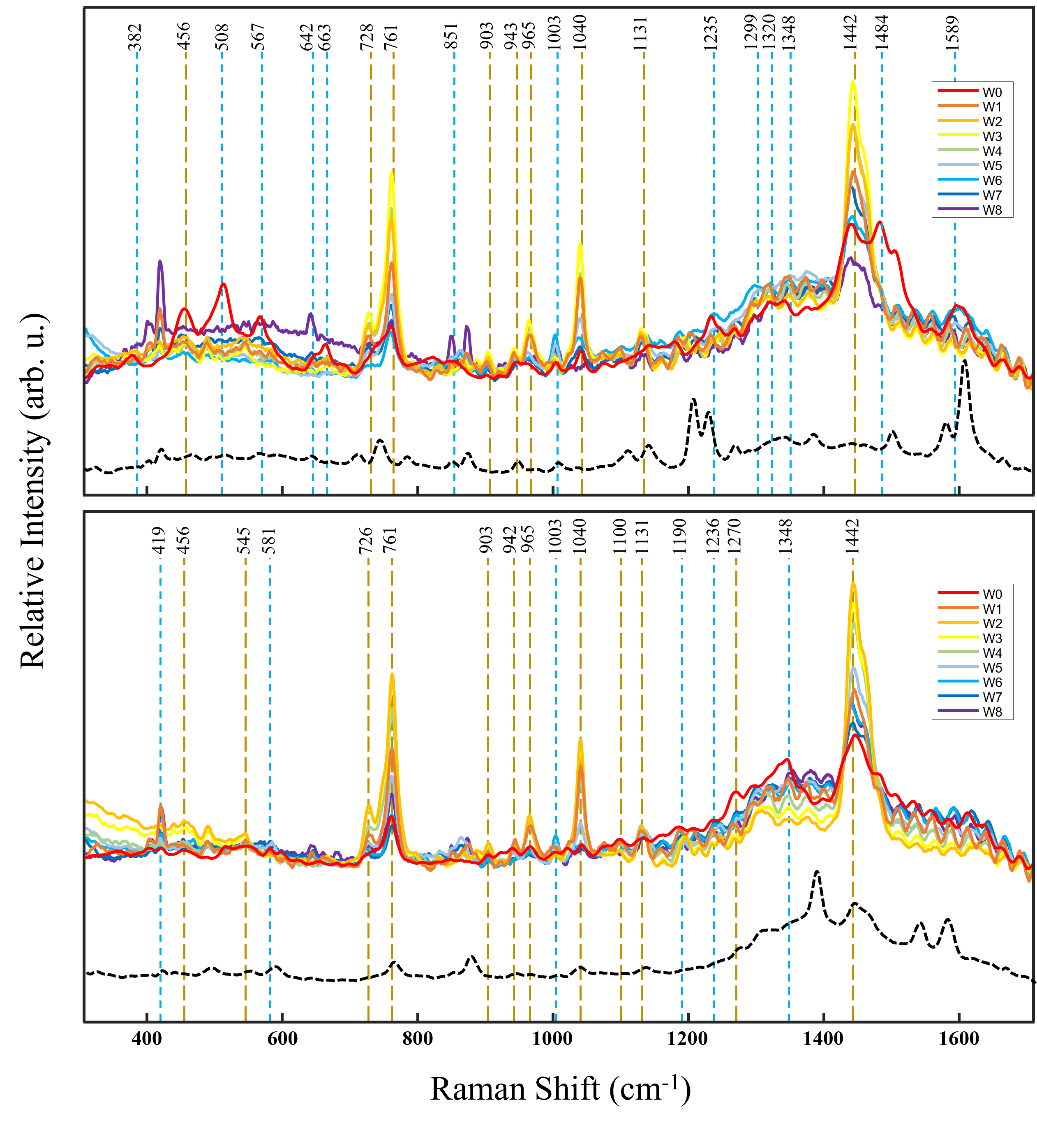


**Figure S7.** Averaged SERS spectra from hair colored with PPU (top) and PRD (bottom), buried in combined soils (Soil types A-C). Gold dashed lines represent bands likely promoted by AuNPs and blue dashed lines represent all other bands from the colorant.


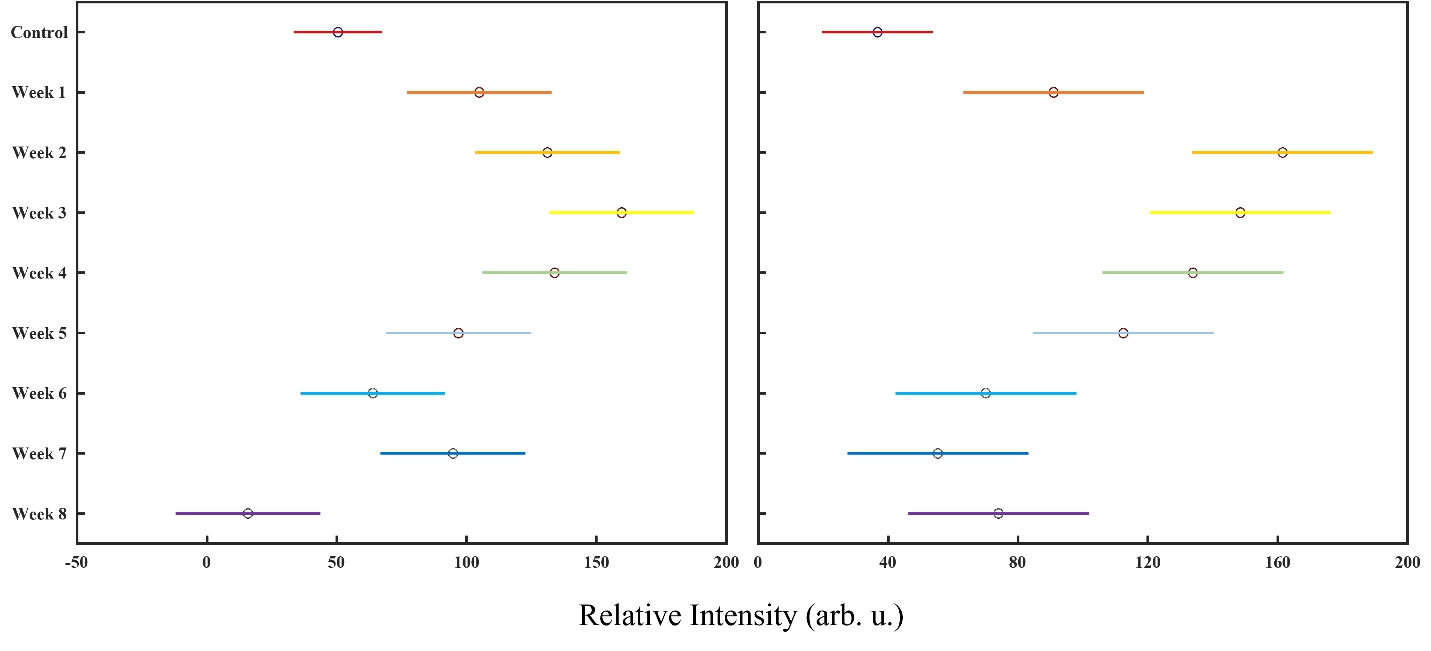


**Figure S8.** Kruskal-Wallis ANOVA multiple comparison graphs of relative intensities in spectra acquired from hair dyed with PPU at 1442 cm^-1^ (left) (AuNP band) and PRD at 1442 cm^-1^ (right) (AuNP band). The solid-colored bars represent 95% confidence intervals for each class and the black circles represent mean values.
